# Prescription regularity and pharmacodynamics mechanism of traditional Chinese medicine in autoimmune hepatitis: A data mining and network pharmacology study

**DOI:** 10.1097/MD.0000000000041146

**Published:** 2024-12-27

**Authors:** Di Guo, Xin Li, Shiya Wei, Fenqing Cai, Yang Liu

**Affiliations:** a College of Basic Medical Sciences, Shanxi University of Chinese Medicine, Jinzhong, PR China; b Basic Laboratory of Integrated Traditional Chinese and Western Medicine, Shanxi University of Chinese Medicine, Jinzhong, PR China; c Engineering Research Center of Cross Innovation for Chinese Traditional Medicine of Shanxi Province, Jinzhong, PR China.

**Keywords:** autoimmune hepatitis, core drug, data mining, network pharmacology, traditional Chinese medicine

## Abstract

This study aims to provide a basis and reference for traditional Chinese medicine (TCM) in treating autoimmune hepatitis (AIH) by exploring the prescription patterns of traditional Chinese medicine (TCM) in treating autoimmune hepatitis (AIH) and predicting the potential mechanisms of core TCM formula. Literature on AIH treated with TCM for was retrieved from WANFANG DATA, China national knowledge infrastructure, and CQVIP databases. The herbals listed in all the prescriptions were analyzed for frequency, correlation, association, and clustering, to filter out the core TCM formula for treating AIH. The core herbals included in the core TCM formula were selected to construct an intersection target network of core herbals-active ingredients-disease-related targets. Gene ontology enrichment and KEGG pathway enrichment analyses were then conducted to reveal the potential mechanism of the core TCM formula in treating AIH. A total of 122 Chinese herbal compound prescriptions involving 196 Chinese herbals were included in this study. These herbals were mostly sweet or bitter in taste, cold in property, and restoring the liver and spleen meridian. Based on the results of frequency, correlation rules, and clustering, the combination of “*Atractylodes macrocephala Koidz.*-*Glycyrrhizae Radix et Rhizoma*-*Cynanchum otophyllum Schneid*-*Bupleuri Radix*-*Poria cocos (Schw.) Wolf.*” was considered as the core TCM formula in treat AIH, which contains 113 active ingredients (including quercetin, kaempferol, naringenin, licochalcone A, and formononetin) and 138 AIH-related targets (involving TP53, AKT1, JUN, STAT3). Moreover, the targets regulated by the core TCM formula are mainly enriched in the biological processes, such as cellular response to lipids, response to inorganic substances, response to hormones, and IL-17 signaling pathway, TNF signaling pathway, and PI3K-Akt signaling pathway. The core TCM formula “*Atractylodes macrocephala Koidz.*-*Glycyrrhizae Radix et Rhizoma*-*Cynanchum otophyllum Schneid*-*Bupleuri Radix*-*Poria cocos (Schw.) Wolf.*” may have a good potential in treating AIH and is worthy of exploring further to develop innovative drugs for this disorder.

## 1. Introduction

Autoimmune hepatitis (AIH) is a kind of chronic liver disease with unknown etiology. It is characterized by the interface hepatitis in histology, and the presence of autoantibodies, high levels of aminotransferase, and γ-globulins in serology. AIH affects individuals across all age groups worldwide, with a higher incidence in females.^[1]^ A meta-analysis revealed the global annual incidence rate and prevalence rate of AIH is 1.37 per 100,000 people (1.11 in females and 0.22 in males) and 17.44 per 100,000 people (12.77 in females and 2.91 in males).^[2]^ The primary treatment for active AIH is immunosuppressive therapy, especially a combination of azathioprine and corticosteroids. This treatment strategy has greatly improved the survival rate of patients with AIH. However, azathioprine treatment also faces challenges, because nearly 1 in 5 patients who receive this treatment report a range of adverse effects, including nausea, vomiting, joint pain, rashes, and fever. These side effects are so severe that about 15% of patients stop azathioprine treatment within the first year of treatment.^[3]^ Therefore, effective, safe, and practical therapy for AIH needs to be explored.

**Figure 1.**
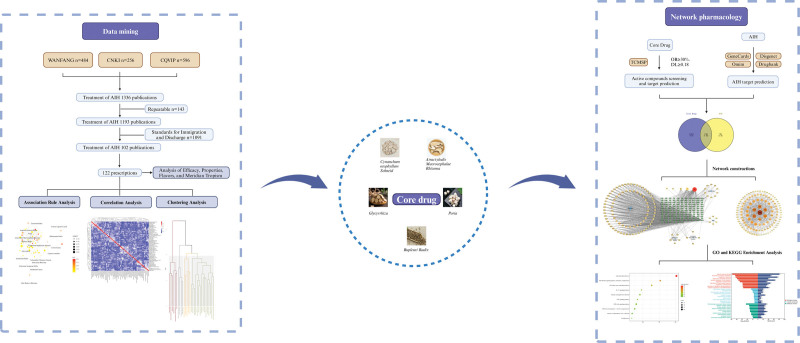
Flowchart of the current research study. The left panel illustrates the data mining process, including the sources and methods of data extraction and processing, leading to the identification of core drugs, displayed in the central section. The right panel details the network pharmacology analysis of the core drugs, elucidating their mechanisms in treating AIH. The entire workflow is depicted with interconnected arrows. AIH = autoimmune hepatitis.

According to the theory of Chinese medicine, AIH belongs to the categories of “hypochondriac pain and “jaundice.”^[4]^ There are thousands of years of experience in traditional Chinese medicine (TCM) for treating AIH, and it has good clinical efficacy, especially for patients with mild and moderate AIH. TCM therapy has the advantages of multi-level, multi-component and multi-target, and its unique treatment methods and concepts have gradually attracted attention, making it the focus and hot field of research in pursuit of better therapeutic effects on AIH. However, the prescription regularity and pharmacodynamics mechanism of TCM for the treatment of AIH is not yet entirely clear. In recent years, the advancements in data mining techniques and network pharmacology have provided help for a more in-depth understanding of the mechanism of TCM treatment of diseases. Thus, in this article, the type, properties, and meridian tropism of TCM used to treat AIH were analyzed with data mining techniques and network pharmacology, moreover, the association rules and clustering relation of these TCMs, and the prescription regularity and pharmacodynamics mechanism of high-frequency herb pairs were also explored, to provide a more scientific and effective basis for the treatment of AIH by TCM. The flowchart is shown in Figure 1.

## 2. Materials and methods

Since the present study did not involve human participants or animals, ethical approval was not required.

### 2.1. Data sources

All literature on oral TCM treatments for AIH from the inception of the databases to May 2023 was collected from 3 major Chinese databases: WANFANG DATA, China National Knowledge Infrastructure (CNKI), and CQVIP. The search string in CNKI was: SU= “autoimmune hepatitis” + “AIH” + “self-immune liver” AND SU=’Chinese medicine’ + “TCM” + “integrated traditional and Western medicine” + “medical cases” + “verified cases” + “Chinese herbal medicine” + “clinical experience” + “experience” + “individual cases” + “medical records” + “case reports” + “Chinese medicinal drugs.” The search string in WANFANG DATA was: M= (“autoimmune hepatitis” or “AIH” or “self-immune liver”) and (“Chinese medicine” or “TCM” or “integrated traditional and Western medicine” or “medical cases” or “verified cases” or “Chinese herbal medicine” or “clinical experience” or “experience” or “individual cases” or “medical records” or “case reports” or “Chinese medicinal drugs”). The VIP search string in CQVIP was: U = (autoimmune hepatitis or AIH or self-immune liver) AND U = (Chinese medicine or TCM or integrated traditional and Western medicine or medical cases or verified cases or Chinese herbal medicine or clinical experience or experience or individual cases or medical records or case reports or Chinese medicinal drugs).

### 2.2. Prescription selection

#### 2.2.1. Inclusion criteria:

Clinical trial journal articles, Master and Doctoral dissertations, individual case studies, and clinical experience literature involving oral administration of Chinese herbal decoction or integrated TCM and Western medicine treatments were collected into this study. The composition of Chinese herbal and the effectiveness of the treatment need to be identified in all literature.

#### 2.2.2. Exclusion criteria:

Review, animal experiments, clinical trials with non-oral administration of Chinese herbal decoction, literature that does not list formula ingredients, clinical trials with unclear or ineffective outcomes, and clinical trials on AIH combined with other diseases were excluded from this study.

### 2.3. Data processing

All the relevant literature was imported into the NoteExpress literature management tool based on the predetermined inclusion and exclusion criteria and the eligible Chinese medicine prescription information extracted from these records was entered into Excel 2016 to create a detailed database encompassing literature title, prescription name, Chinese herbal formulas, and other information such as the properties and meridian tropism of the TCMs.

### 2.4. Standardization of Chinese medicine terminology

According to the Chinese pharmacopoeia (2020 edition),^[5]^ Chinese pharmacy (the textbook for the 14th five-year plan),^[6]^ and Chinese Materia Medica,^[7]^ we have unified and standardized the names of Chinese Materia Medica involved in this study. We also unified the names of the Chinese herbals whose processing methods had little influence on the efficacy and meridian tropism. Moreover, different processing methods of the same Chinese herbals were classified under a single name. All aspects of the Chinese herbals with multiple properties and meridian tropism were fully recorded.

### 2.5. Data analysis

In the context of R language software (version 4.3.1), we conducted in-depth data mining on the TCM prescription data to identify key attributes of Chinese Materia Medica such as property, taste, efficacy, compatibility, and prescription composition. The most important Chinese herbals were also identified. First, the corrplot function was used to conduct correlation analysis on the data set, and the drug combinations with clinical therapeutic effects were screened out. Then, association rule analysis was performed with the “arules” package to explore compatibility patterns for core Chinese herbals, with a minimum support of 0.12 and a minimum confidence of 0.8, and a network diagram was used to visualize the results. Finally, the hierarchical clustering analysis of frequently used Chinese herbals was implemented with the “cluster” package to identify core prescription regularity. The Euclidean distance was chosen as the metric, and a dendrogram was constructed with the Ward D method.

### 2.6. Network pharmacology analysis

#### 2.6.1. Core components screening and target prediction:

The TCM systems pharmacology database (TCMSP, https://www.tcmsp-e.com/#/database)^[8]^was utilized to obtain all active ingredients of the core Chinese herbals. Based on the criteria of oral bioavailability (OB) ≥ 30% and drug-likeness (DL) ≥ 0.18, effective components were screened. Then, the TCMSP database was used to retrieve the targets of these active compounds, and according to the UniProt database (https://www.uniprot.org/),^[9]^ the targets were converted into corresponding gene items. After removing the duplicates, we obtained the gene targets of the drug actions.

#### 2.6.2. AIH targets filtration:

Using “autoimmune hepatitis” as the keyword, we searched for AIH-related pathogenic targets in the following 4 databases: GeneCards (https://www.genecards.org),^[10]^ Omim (http://www.omim.org),^[11]^ Disgenet (https://www.disgenet.org),^[12]^ and Drugbank (https://go.drugbank.com).^[13]^ In particular, in the GeneCards database, targets with scores >2 times the median were selected as relevant targets for AIH. The AIH-related targets list was constructed after consolidating the targets from these 4 databases and eliminating the duplicates.

#### 2.6.3. Construction of intersection target network of core Chinese herbals and active ingredients:

The Venny 2.1.0 tool (https://bioinfogp.cnb.csic.es/tools/venny/)^[14]^ was used to create Venn diagrams of the Chinese herbal targets and disease targets and identify the intersection targets. To visualize this information more intuitively, The Cytoscape 3.9.1 software^[15]^ was performed to visualize the intersection targets and create a network graph of the intersection target network of core Chinese herbals and active ingredients.

#### 2.6.4. Construction of PPI network:

To further explore the relationships between these intersection targets, we used the STRING database (https://string-db.org/)^[16]^ to construct their protein–protein interaction (PPI) network. During this process, we selected “homo sapiens” as the biological species and set the minimum interaction threshold to “high confidence (0.900),” as well as hid the disconnected proteins.

#### 2.6.5. GO functional enrichment analysis and KEGG pathway enrichment analysis:

To better elucidate the roles of these targets in gene functions and signaling pathways and reveal the multi-mechanistic actions of active ingredients of core herbals from a systematic perspective, we also performed the gene ontology (GO) functional enrichment analysis and Kyoto encyclopedia of genes and genomes (KEGG) pathway enrichment analysis on the potential targets for the treatment of AIH with TCM by using the Metascape database (https://metascape.org/gp/index.html#/main/step1). The Weishengxin online graphing platform was utilized to visualize the results.

## 3. Results

### 3.1. Literature inclusion and frequency analysis of Chinese herbals

In the preliminary literature search, 1336 relevant documents were retrieved. After screening with the predetermined inclusion and exclusion criteria, 102 articles concerning the treatment of AIH with TCM were ultimately selected, from which we extracted 122 TCM prescriptions. A total of 122 TCM prescriptions and 196 different Chinese herbals were included in this study. The cumulative frequency of all herbals was 1602 times. Among them, the use frequency of 31 kinds of Chinese herbals was more than 15 times, and the total frequency of selection was 1039 times, accounting for 64.9% (shown in Table 1). The top ten most frequently used Chinese herbals were *Bupleuri Radix, Cynanchum otophyllum Schneid, Glycyrrhiza, Artemisiae Scopariae Herba, Rehmanniae Radix, Poria, Atractylodis Macrocephalae Rhizoma, Angelicae Sinensis Radix, Paeoniae Rubra Radix, Curcumae Radix*.

**Table 1:** The frequency ranking of the herbs occurred ≥ 15 times.

Serial number	Drug	Frequency count	Frequency 1	Frequency 2
1	*Bupleuri Radix*	72	4.49%	59.02%
2	*Cynanchum otophyllum Schneid*	68	4.24%	55.74%
3	*Glycyrrhiza*	67	4.18%	54.92%
4	*Artemisiae Scopariae Herba*	61	3.81%	50.00%
5	*Rehmanniae Radix*	51	3.18%	41.80%
6	*Poria*	50	3.12%	40.98%
7	*Atractylodis Macrocephalae Rhizoma*	46	2.87%	37.70%
8	*Angelicae Sinensis Radix*	45	2.81%	36.89%
9	*Paeoniae Rubra Radix*	43	2.68%	35.25%
10	*Curcumae Radix*	40	2.50%	32.79%
11	*Salvia miltiorrhiza*	39	2.43%	31.97%
12	*Moutan Cortex*	37	2.31%	30.33%
13	*Astragali Radix*	35	2.18%	28.69%
14	*Gardeniae Fructus*	35	2.18%	28.69%
15	*Scutellariae Radix*	26	1.62%	21.31%
16	*Citri Reticulatae Pericarpium*	25	1.56%	20.49%
17	*Schisandrae Chinensis Fructus*	25	1.56%	20.49%
18	*Rhei Radix et Rhizoma*	23	1.44%	18.85%
19	*Dioscoreae Rhizoma*	22	1.37%	18.03%
20	*Coicis Semen*	22	1.37%	18.03%
21	*Alismatis Rhizoma*	22	1.37%	18.03%
22	*Lycii Fructus*	21	1.31%	17.21%
23	*Ophiopogonis Radix*	21	1.31%	17.21%
24	*Rubiae Radix et Rhizoma*	21	1.31%	17.21%
25	*Chuanxiong Rhizoma*	20	1.25%	16.39%
26	*Fructus Ligustri Lucidi*	18	1.12%	14.75%
27	*Corni Fructus*	18	1.12%	14.75%
28	*Juglandis Semen*	18	1.12%	14.75%
29	*Cyperus rotundus*	17	1.06%	13.93%
30	*Sedi Herba*	16	1.00%	13.11%
31	*Glehniae Radix*	15	0.94%	12.30%

Note: frequency 1 refers to the proportion of medicinal frequency of a single herb in the total drug frequency, frequency 2 refers to the proportion of the medicinal frequency of a single herb in the number of prescriptions.

### 3.2. Efficacy analysis of Chinese herbals

According to their efficacy, the 196 kinds of Chinese Herbals were categorized into 18 classes, and the total frequency of selection was 1587 times (shown in Table 2). Tonic drugs were used the most frequently, with the highest frequency of 434 times, accounting for 27.35%, followed by heat-clearing drugs with a frequency of 261 times and damp-clearing drugs with a frequency of 225 times.

**Table 2:** Statistics on the efficacy of 196 TCMs.

Efficacy	Frequency count	Efficacy	Frequency count
Tonic medicines	434	Wind-eliminating and dampness-resolving medicines	33
Interior heat-clearing medicines	261	Dampness-transforming medicines	30
Urination-promoting and dampness-draining medicines	225	Purgative medicines	25
Blood-circulating and blood stasis-resolving medicines	168	Tranquillizing medicines	22
Exterior-releasing medicines	110	Cough-stopping and panting-alleviating medicines	15
Qi-regulating medicines	101	Interior-warming medicines	11
Astringing medicines	53	Liver-soothing and wind-extinguishing medicines	11
Digestion-promoting medicines	47	Orifice-opening medicines	3
Haemostatic medicines	36	Parasite-expelling medicines	2

### 3.3. Analysis of properties, flavors, and meridian tropism

As exhibited in Tables 3 to 5, according to the classification of properties, for these 196 kinds of Chinese Herbals, cold-property medicines accounted for 47.13% (755 times) and warm-property medicines accounted for 25.16% (403 times). According to the classification of drug taste, bitter flavor accounted for 34.71% (897 times) and sweet flavor accounted for 30.34% (785 times). Based on the classification of meridian tropism, the Liver Meridian of Foot-Jueyin (LR) was accounted for 20.89% of the instances (946 times), and the Spleen Meridian of Foot-Taiyin (SP) accounted for 16.54% of the instances (749 times).

**Table 3:** Frequency analysis of medicinal properties in the 196 TCMs.

Drug taste	Frequency count	Frequency
Bland flavor	124	4.80%
Sweet flavor	784	30.34%
Sour flavor	174	6.73%
Pungent flavor	525	20.32%
Bitter flavor	897	34.71%
Astringent flavor	32	1.24%

**Table 4:** The flavor and taste of the 196 TCMs.

Meridian tropism	Frequency count	Frequency
The lung meridian of hand-Taiyin(LU)	642	14.18%
The large intestine meridian of hand-Yangming (LI)	115	2.54%
The stomach meridian of foot-Yangming(ST)	502	11.08%
The spleen meridian of foot-Taiyin (SP)	749	16.54%
The heart meridian of hand-Shaoyin(HT)	525	11.59%
The small intestine meridian of hand-Taiyang (SI)	103	2.27%
The bladder meridian of foot-Taiyang (BL)	117	2.58%
The kidney meridian of foot-Shaoyin (SI)	422	9.32%
The pericardium meridian of hand-Jueyin (PC)	48	1.06%
The Sanjiao meridian of hand-Shaoyang (TE)	58	1.28%
The gallbladder meridian of foot-Shaoyang (GB)	302	6.67%
The liver meridian of foot-Jueyin (LR)	946	20.89%

**Table 5:** The meridian tropism of the 196 TCMs.

Medicinal properties	Frequency count	Frequency
Cold-property medicines	755	47.13%
Warm-property medicines	403	25.16%
Neutral-property medicines	358	22.35%
Cool-property medicines	75	4.68%
Heat-property medicines	11	0.69%

### 3.4. Correlation analysis of herb pairs

**Figure 2.**
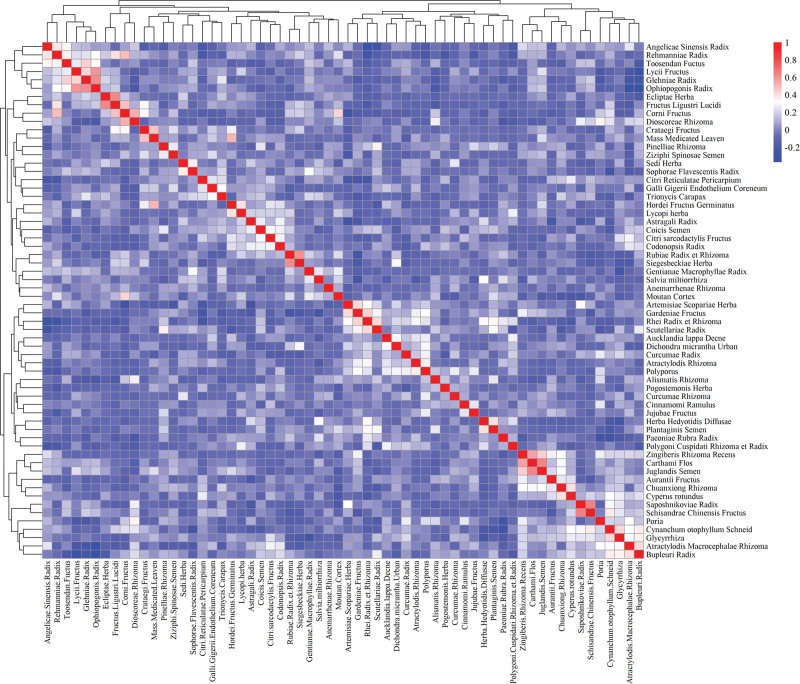
Pearson correlation coefficient of TCM used more than 5 times. Pearson correlation analysis was performed on 62 TCMs that were used more than 5 times out of 196 TCMs. The red color represents the positive correlation and the blue color represents the negative correlation. TCM = traditional Chinese medicine.

In line with the use frequency of the 196 kinds of Chinese herbals, 62 kinds of herbals used more than 5 times were selected to analyze the correlation of herb pairs with the Pearson Correlation Analysis method, and the result was visualized with the corrplot function of the R language software (shown in Fig. 2). Based on the correlation coefficients, we ranked all drug pairs and listed the top 20 with the highest correlation coefficients in Table 6.

**Table 6:** Correlation coefficients of top 20 drug pairs used more than 5 times.

Serial number	Drug-pair	Correlation coefficient
1	*Schisandrae Chinensis Fructus-Saposhnikoviae Radix*	0.65
2	*Juglandis Semen-Carthami Flos*	0.64
3	*Fructus Ligustri Lucidi-Ecliptae Herba*	0.64
4	*Ophiopogonis Radix-Glehniae Radix*	0.62
5	*Rubiae Radix et Rhizoma-Siegesbeckiae Herba*	0.61
6	*Lycii Fructus-Ophiopogonis Radix*	0.60
7	*Carthami Flos-Zingiberis Rhizoma Recens*	0.56
8	*Dioscoreae Rhizoma-Corni Fructus*	0.53
9	*Hordei Fructus Germinatus-Mass Medicated Leaven*	0.51
10	*Rehmanniae Radix-Corni Fructus*	0.49
11	*Corni Fructus-Fructus Ligustri Lucidi*	0.48
12	*Crataegi Fructus-Mass Medicated Leaven*	0.47
13	*Juglandis Semen-Zingiberis Rhizoma Recens*	0.44
14	*Glehniae Radix-Toosendan Fuctus*	0.44
15	*Gardeniae Fructus-Rhei Radix et Rhizoma*	0.44
16	*Moutan Cortex-Corni Fructus*	0.43
17	*Lycii Fructus-Glehniae Radix*	0.42
18	*Cynanchum otophyllum Schneid-Glycyrrhiza*	0.42
19	*Artemisiae Scopariae Herba-Gardeniae Fructus*	0.42
20	*Scutellariae Radix-Rhei Radix et Rhizoma*	0.41

### 3.5. Association rule analysis

**Figure 3.**
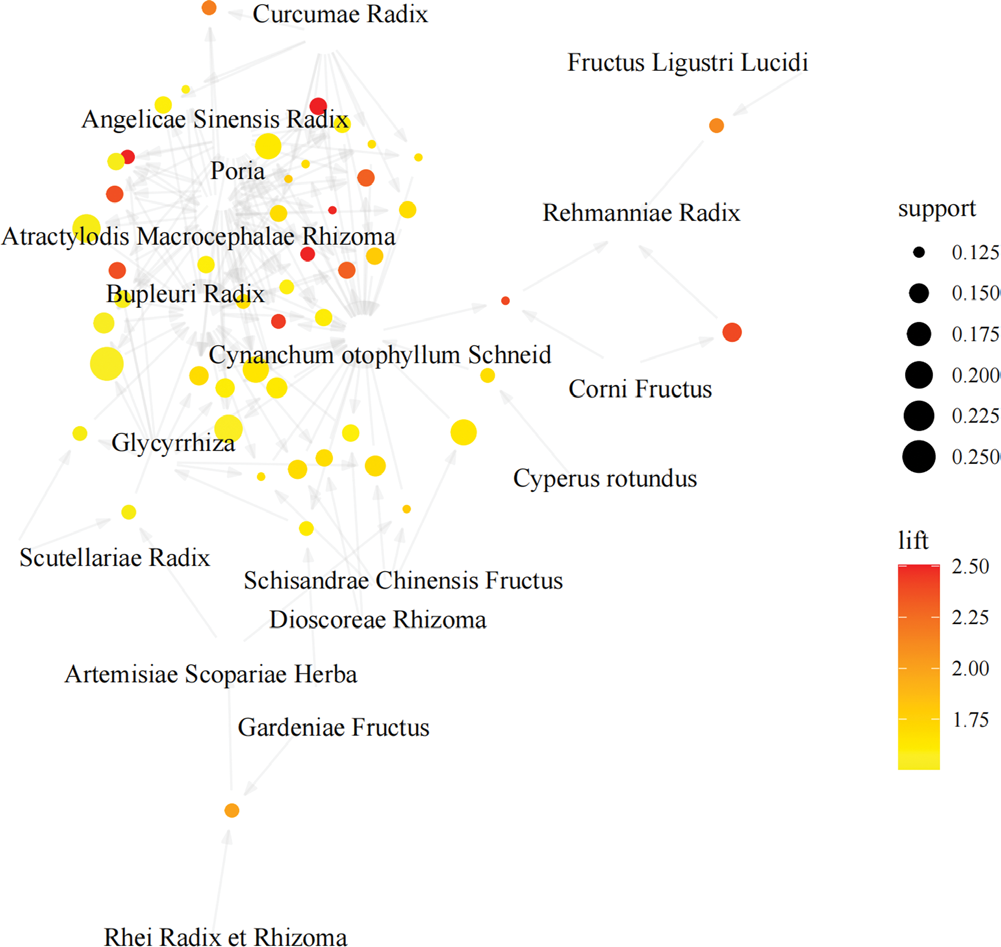
Network diagram of association rule analysis of high-frequency TCMs. The Apriori algorithm was applied to 196 TCMs to identify high-frequency combinations for treating AIH. With a minimum support degree of 0.12 and confidence of 0.8, 118 significant association rules were found. Bubble size represents support degree, color indicates lift value and arrow count shows TCM pairing frequency. AIH = autoimmune hepatitis, TCM = traditional Chinese medicine.

With the Apriori function, we conducted the association rule analysis on the 196 Chinese herbals to explore the high-frequency drug combinations for treating AIH. As the minimum support degree is set to 0.12 and the minimum confidence degree is set to 0.8, a total of 118 core drug-pair association rules are obtained, and the enhancement degree of association rules is >1, which means that all the rules are valid. Among the 118 rules, there are 14 association rules for 2 categories of drug-pairing, 37 association rules for 3 categories of drug pairing, 56 association rules for 4 categories of drug pairing, and 11 association rules for 5 kinds of drug pairing (exhibited in Tables 7–10). The top 50 association rules with the highest support degree were visualized using the arules and arulesViz functions of R language software (Fig. 3). The bubble size represents the support degree of the drug pair, color intensity signifies the lifting degree, and the more arrows pointed, the more often the drug is involved in pairing.

**Table 7:** Binomial association rules of TCMs in 122 prescriptions.

Serial number	Drug combinations	Support	Confidence
1	{*Atractylodis Macrocephalae Rhizoma*}=>{*Bupleuri Radix*}	0.32	0.85
2	{*Gardeniae Fructus*}=>{*Artemisiae Scopariae Herba*}	0.24	0.83
3	{*Schisandrae Chinensis Fructus*}=>{*Cynanchum otophyllum Schneid*}	0.19	0.92
4	{*Scutellariae Radix*}=>{*Bupleuri Radix*}	0.18	0.85
5	{*Schisandrae Chinensis Fructus*}=>{*Glycyrrhiza*}	0.16	0.8
6	{*Rhei Radix et Rhizoma*}=>{*Artemisiae Scopariae Herba*}	0.16	0.83
7	{*Corni Fructus*}=>{*Rehmanniae Radix*}	0.15	1
8	{*Dioscoreae Rhizoma*}=>{*Cynanchum otophyllum Schneid*}	0.15	0.82
9	{*Dioscoreae Rhizoma*}=>{*Bupleuri Radix*}	0.15	0.82
10	{*Fructus Ligustri Lucidi*}=>{*Rehmanniae Radix*}	0.13	0.89
11	{*Cyperus rotundus*}=>{*Cynanchum otophyllum Schneid*}	0.13	0.94
12	{*Corni Fructus*}=>{*Cynanchum otophyllum Schneid*}	0.12	0.83
13	{*Cyperus rotundus*}=>{*Glycyrrhiza*}	0.12	0.88
14	{*Cyperus rotundus*}=>{*Bupleuri Radix*}	0.12	0.88

**Table 8:** Trinomial association rules of TCMs in 122 prescriptions.

Serial number	Drug combinations	Support	Confidence
1	{*Atractylodis Macrocephalae Rhizoma,Cynanchum otophyllum Schneid*}*=>*{*Bupleuri Radix*}	0.25	0.89
2	{*Atractylodis Macrocephalae Rhizoma,Glycyrrhiza*}*=>*{*Bupleuri Radix*}	0.25	0.91
3	{*Bupleuri Radix,Poria*}*=>*{*Cynanchum otophyllum Schneid*}	0.24	0.85
4	{*Cynanchum otophyllum Schneid,Poria*}*=>*{*Bupleuri Radix*}	0.24	0.85
5	{*Glycyrrhiza,Poria*}*=>*{*Cynanchum otophyllum Schneid*}	0.22	0.82
6	{*Artemisiae Scopariae Herba,Cynanchum otophyllum Schneid*}*=>*{*Glycyrrhiza*}	0.21	0.81
7	{*Atractylodis Macrocephalae Rhizoma,Poria*}*=>*{*Bupleuri Radix*}	0.20	0.89
8	{*Atractylodis Macrocephalae Rhizoma,Poria*}*=>*{*Cynanchum otophyllum Schneid*}	0.20	0.86
9	{*Curcumae Radix,Cynanchum otophyllum Schneid*}*=>*{*Glycyrrhiza*}	0.18	0.85
10	{*Angelicae Sinensis Radix,Glycyrrhiza*}*=>*{*Cynanchum otophyllum Schneid*}	0.17	0.81
11	{*Bupleuri Radix,Curcumae Radix*}*=>*{*Glycyrrhiza*}	0.17	0.84
12	{*Artemisiae Scopariae Herba,Atractylodis Macrocephalae Rhizoma*}*=>*{*Bupleuri Radix*}	0.16	0.80
13	{*Bupleuri Radix,Curcumae Radix*}*=>*{*Cynanchum otophyllum Schneid*}	0.16	0.80
14	{*Angelicae Sinensis Radix,Atractylodis Macrocephalae Rhizoma*}*=>*{*Bupleuri Radix*}	0.16	0.83
15	{*Cynanchum otophyllum Schneid,Schisandrae Chinensis Fructus*}*=>*{*Glycyrrhiza*}	0.16	0.83
16	{*Angelicae Sinensis Radix,Poria*}*=>*{*Atractylodis Macrocephalae Rhizoma*}	0.16	0.86
17	{*Angelicae Sinensis Radix,Poria*}*=>*{*Bupleuri Radix*}	0.16	0.86
18	{*Glycyrrhiza,Schisandrae Chinensis Fructus*}*=>*{*Cynanchum otophyllum Schneid*}	0.16	0.95
19	{*Bupleuri Radix,Schisandrae Chinensis Fructus*}*=>*{*Cynanchum otophyllum Schneid*}	0.15	0.95
20	{*Angelicae Sinensis Radix,Poria*}*=>*{*Cynanchum otophyllum Schneid*}	0.15	0.82
21	{*Atractylodis Macrocephalae Rhizoma,Curcumae Radix*}*=>*{*Cynanchum otophyllum Schneid*}	0.14	0.94
22	{*Cynanchum otophyllum Schneid,Dioscoreae Rhizoma*}*=>*{*Bupleuri Radix*}	0.14	0.94
23	{*Atractylodis Macrocephalae Rhizoma,Curcumae Radix*}*=>*{*Bupleuri Radix*}	0.14	0.94
24	{*Bupleuri Radix,Dioscoreae Rhizoma*}*=>*{*Cynanchum otophyllum Schneid*}	0.14	0.94
25	{*Gardeniae Fructus,Rhei Radix et Rhizoma*}*=>*{*Artemisiae Scopariae Herba*}	0.13	1.00
26	{*Astragali Radix,Glycyrrhiza*}*=>*{*Bupleuri Radix*}	0.13	0.80
27	{*Glycyrrhiza,Schisandrae Chinensis Fructus*}*=>*{*Bupleuri Radix*}	0.13	0.80
28	{*Atractylodis Macrocephalae Rhizoma,Curcumae Radix*}*=>*{*Poria*}	0.13	0.89
29	{*Bupleuri Radix,Schisandrae Chinensis Fructus*}*=>*{*Glycyrrhiza*}	0.13	0.84
30	{*Artemisiae Scopariae Herba,Rhei Radix et Rhizoma*}*=>*{*Gardeniae Fructus*}	0.13	0.84
31	{*Artemisiae Scopariae Herba,Scutellariae Radix*}*=>*{*Bupleuri Radix*}	0.13	0.89
32	{*Glycyrrhiza,Scutellariae Radix*}*=>*{*Bupleuri Radix*}	0.13	0.89
33	{*Cynanchum otophyllum Schneid,Gardeniae Fructus*}*=>*{*Glycyrrhiza*}	0.13	0.89
34	{*Corni Fructus,Rehmanniae Radix*}*=>*{*Cynanchum otophyllum Schneid*}	0.12	0.83
35	{*Angelicae Sinensis Radix,Curcumae Radix*}*=>*{*Cynanchum otophyllum Schneid*}	0.12	0.94
36	{*Artemisiae Scopariae Herba,Schisandrae Chinensis Fructus*}*=>*{*Cynanchum otophyllum Schneid*}	0.12	1.00
37	{*Corni Fructus,Cynanchum otophyllum Schneid*}*=>*{*Rehmanniae Radix*}	0.12	1.00

**Table 9:** Quaternary association rules of TCMs in 122 prescriptions.

Serial number	Drug combinations	Support	Confidence
1	{*Atractylodis Macrocephalae Rhizoma,Bupleuri Radix,Cynanchum otophyllum Schneid*}*=>*{*Glycyrrhiza*}	0.20	0.81
2	{*Atractylodis Macrocephalae Rhizoma,Bupleuri Radix,Glycyrrhiza*}*=>*{*Cynanchum otophyllum Schneid*}	0.20	0.81
3	{*Atractylodis Macrocephalae Rhizoma,Cynanchum otophyllum Schneid,Glycyrrhiza*}*=>*{*Bupleuri Radix*}	0.20	0.93
4	{*Atractylodis Macrocephalae Rhizoma,Cynanchum otophyllum Schneid,Poria*}*=>*{*Bupleuri Radix*}	0.19	0.96
5	{*Atractylodis Macrocephalae Rhizoma,Bupleuri Radix,Poria*}*=>*{*Cynanchum otophyllum Schneid*}	0.19	0.92
6	{*Bupleuri Radix,Glycyrrhiza,Poria*}*=>*{*Cynanchum otophyllum Schneid*}	0.18	0.88
7	{*Cynanchum otophyllum Schneid,Glycyrrhiza,Poria*}*=>*{*Bupleuri Radix*}	0.18	0.81
8	{*Atractylodis Macrocephalae Rhizoma,Glycyrrhiza,Poria*}*=>*{*Bupleuri Radix*}	0.16	0.90
9	{*Atractylodis Macrocephalae Rhizoma,Glycyrrhiza,Poria*}*=>*{*Cynanchum otophyllum Schneid*}	0.16	0.90
10	{*Artemisiae Scopariae Herba,Bupleuri Radix,Cynanchum otophyllum Schneid*}*=>*{*Glycyrrhiza*}	0.15	0.82
11	{*Atractylodis Macrocephalae Rhizoma,Curcumae Radix,Cynanchum otophyllum Schneid*}*=>*{*Bupleuri Radix*}	0.14	1.00
12	{*Angelicae Sinensis Radix,Bupleuri Radix,Poria*}*=>*{*Cynanchum otophyllum Schneid*}	0.14	0.89
13	{*Angelicae Sinensis Radix,Atractylodis Macrocephalae Rhizoma,Glycyrrhiza*}*=>*{*Bupleuri Radix*}	0.14	0.89
14	{*Angelicae Sinensis Radix,Atractylodis Macrocephalae Rhizoma,Bupleuri Radix*}*=>*{*Glycyrrhiza*}	0.14	0.85
15	{*Angelicae Sinensis Radix,Atractylodis Macrocephalae Rhizoma,Bupleuri Radix*}*=>*{*Poria*}	0.14	0.85
16	{*Atractylodis Macrocephalae Rhizoma,Bupleuri Radix,Curcumae Radix*}*=>*{*Cynanchum otophyllum Schneid*}	0.14	1.00
17	{*Angelicae Sinensis Radix,Bupleuri Radix,Glycyrrhiza*}*=>*{*Atractylodis Macrocephalae Rhizoma*}	0.14	0.89
18	{*Angelicae Sinensis Radix,Atractylodis Macrocephalae Rhizoma,Poria*}*=>*{*Cynanchum otophyllum Schneid*}	0.14	0.89
19	{*Angelicae Sinensis Radix,Bupleuri Radix,Poria*}*=>*{*Atractylodis Macrocephalae Rhizoma*}	0.14	0.89
20	{*Angelicae Sinensis Radix,Atractylodis Macrocephalae Rhizoma,Poria*}*=>*{*Bupleuri Radix*}	0.14	0.89
21	{*Angelicae Sinensis Radix,Bupleuri Radix,Cynanchum otophyllum Schneid*}*=>*{*Poria*}	0.14	0.94
22	{*Angelicae Sinensis Radix,Cynanchum otophyllum Schneid,Poria*}*=>*{*Bupleuri Radix*}	0.14	0.94
23	{*Angelicae Sinensis Radix,Cynanchum otophyllum Schneid,Poria*}*=>*{*Atractylodis Macrocephalae Rhizoma*}	0.14	0.94
24	{*Angelicae Sinensis Radix,Atractylodis Macrocephalae Rhizoma,Cynanchum otophyllum Schneid*}*=>*{*Poria*}	0.14	0.94
25	{*Bupleuri Radix,Curcumae Radix,Cynanchum otophyllum Schneid*}*=>*{*Atractylodis Macrocephalae Rhizoma*}	0.14	0.85
26	{*Bupleuri Radix,Curcumae Radix,Cynanchum otophyllum Schneid*}*=>*{*Poria*}	0.13	0.80
27	{*Bupleuri Radix,Curcumae Radix,Cynanchum otophyllum Schneid*}*=>*{*Glycyrrhiza*}	0.13	0.80
28	{*Angelicae Sinensis Radix,Atractylodis Macrocephalae Rhizoma,Bupleuri Radix*}*=>*{*Cynanchum otophyllum Schneid*}	0.13	0.80
29	{*Angelicae Sinensis Radix,Atractylodis Macrocephalae Rhizoma,Glycyrrhiza*}*=>*{*Poria*}	0.13	0.84
30	{*Angelicae Sinensis Radix,Atractylodis Macrocephalae Rhizoma,Poria*}*=>*{*Glycyrrhiza*}	0.13	0.84
31	{*Artemisiae Scopariae Herba,Atractylodis Macrocephalae Rhizoma,Bupleuri Radix*}*=>*{*Glycyrrhiza*}	0.13	0.80
32	{*Angelicae Sinensis Radix,Glycyrrhiza,Poria*}*=>*{*Atractylodis Macrocephalae Rhizoma*}	0.13	0.94
33	{*Artemisiae Scopariae Herba,Atractylodis Macrocephalae Rhizoma,Glycyrrhiza*}*=>*{*Bupleuri Radix*}	0.13	0.89
34	{*Angelicae Sinensis Radix,Bupleuri Radix,Cynanchum otophyllum Schneid*}*=>*{*Atractylodis Macrocephalae Rhizoma*}	0.13	0.89
35	{*Curcumae Radix,Cynanchum otophyllum Schneid,Poria*}*=>*{*Bupleuri Radix*}	0.13	0.89
36	{*Bupleuri Radix,Curcumae Radix,Poria*}*=>*{*Cynanchum otophyllum Schneid*}	0.13	0.89
37	{*Angelicae Sinensis Radix,Atractylodis Macrocephalae Rhizoma,Cynanchum otophyllum Schneid*}*=>*{*Bupleuri Radix*}	0.13	0.89
38	{*Angelicae Sinensis Radix,Glycyrrhiza,Poria*}*=>*{*Bupleuri Radix*}	0.12	0.88
39	{*Artemisiae Scopariae Herba,Atractylodis Macrocephalae Rhizoma,Cynanchum otophyllum Schneid*}*=>*{*Glycyrrhiza*}	0.12	0.83
40	{*Bupleuri Radix,Glycyrrhiza,Schisandrae Chinensis Fructus*}*=>*{*Cynanchum otophyllum Schneid*}	0.12	0.94
41	{*Atractylodis Macrocephalae Rhizoma,Curcumae Radix,Poria*}*=>*{*Cynanchum otophyllum Schneid*}	0.12	0.94
42	{*Atractylodis Macrocephalae Rhizoma,Curcumae Radix,Poria*}*=>*{*Bupleuri Radix*}	0.12	0.94
43	{*Artemisiae Scopariae Herba,Atractylodis Macrocephalae Rhizoma,Cynanchum otophyllum Schneid*}*=>*{*Bupleuri Radix*}	0.12	0.83
44	{*Artemisiae Scopariae Herba,Atractylodis Macrocephalae Rhizoma,Glycyrrhiza*}*=>*{*Cynanchum otophyllum Schneid*}	0.12	0.83
45	{*Angelicae Sinensis Radix,Cynanchum otophyllum Schneid,Poria*}*=>*{*Glycyrrhiza*}	0.12	0.83
46	{*Angelicae Sinensis Radix,Bupleuri Radix,Cynanchum otophyllum Schneid*}*=>*{*Glycyrrhiza*}	0.12	0.83
47	{*Angelicae Sinensis Radix,Glycyrrhiza,Poria*}*=>*{*Cynanchum otophyllum Schneid*}	0.12	0.88
48	{*Bupleuri Radix,Curcumae Radix,Poria*}*=>*{*Glycyrrhiza*}	0.12	0.83
49	{*Curcumae Radix,Cynanchum otophyllum Schneid,Poria*}*=>*{*Glycyrrhiza*}	0.12	0.83
50	{*Bupleuri Radix,Curcumae Radix,Poria*}*=>*{*Atractylodis Macrocephalae Rhizoma*}	0.12	0.83
51	{*Curcumae Radix,Cynanchum otophyllum Schneid,Poria*}*=>*{*Atractylodis Macrocephalae Rhizoma*}	0.12	0.83
52	{*Bupleuri Radix,Cynanchum otophyllum Schneid,Schisandrae Chinensis Fructus*}*=>*{*Glycyrrhiza*}	0.12	0.83
53	{*Atractylodis Macrocephalae Rhizoma,Curcumae Radix,Cynanchum otophyllum Schneid*}*=>*{*Poria*}	0.12	0.88
54	{*Atractylodis Macrocephalae Rhizoma,Bupleuri Radix,Curcumae Radix*}*=>*{*Poria*}	0.12	0.88
55	{*Curcumae Radix,Glycyrrhiza,Poria*}*=>*{*Bupleuri Radix*}	0.12	0.88
56	{*Curcumae Radix,Glycyrrhiza,Poria*}*=>*{*Cynanchum otophyllum Schneid*}	0.12	0.88

**Table 10:** Quinary association rules of TCMs in 122 prescriptions.

Serial number	Drug combinations	Support	Confidence
1	{*Atractylodis Macrocephalae Rhizoma,Cynanchum otophyllum Schneid,Glycyrrhiza,Poria*}*=>*{*Bupleuri Radix*}	0.15	0.95
2	{*Atractylodis Macrocephalae Rhizoma,Bupleuri Radix,Glycyrrhiza,Poria*}*=>*{*Cynanchum otophyllum Schneid*}	0.15	0.95
3	{*Bupleuri Radix,Cynanchum otophyllum Schneid,Glycyrrhiza,Poria*}*=>*{*Atractylodis Macrocephalae Rhizoma*}	0.15	0.82
4	{*Angelicae Sinensis Radix,Atractylodis Macrocephalae Rhizoma,Bupleuri Radix,Cynanchum otophyllum Schneid*}*=>*{*Poria*}	0.13	1.00
5	{*Angelicae Sinensis Radix,Atractylodis Macrocephalae Rhizoma,Cynanchum otophyllum Schneid,Poria*}*=>*{*Bupleuri Radix*}	0.13	0.94
6	{*Angelicae Sinensis Radix,Atractylodis Macrocephalae Rhizoma,Bupleuri Radix,Poria*}*=>*{*Cynanchum otophyllum Schneid*}	0.13	0.94
7	{*Angelicae Sinensis Radix,Bupleuri Radix,Cynanchum otophyllum Schneid,Poria*}*=>*{*Atractylodis Macrocephalae Rhizoma*}	0.13	0.94
8	{*Atractylodis Macrocephalae Rhizoma,Curcumae Radix,Cynanchum otophyllum Schneid,Poria*}*=>*{*Bupleuri Radix*}	0.12	1.00
9	{*Atractylodis Macrocephalae Rhizoma,Bupleuri Radix,Curcumae Radix,Poria*}*=>*{*Cynanchum otophyllum Schneid*}	0.12	1.00
10	{*Bupleuri Radix,Curcumae Radix,Cynanchum otophyllum Schneid,Poria*}*=>*{*Atractylodis Macrocephalae Rhizoma*}	0.12	0.94
11	{*Atractylodis Macrocephalae Rhizoma,Bupleuri Radix,Curcumae Radix,Cynanchum otophyllum Schneid*}*=>*{*Poria*}	0.12	0.88

### 3.6. Clustering analysis of high-frequency Chinese herbals

**Figure 4.**
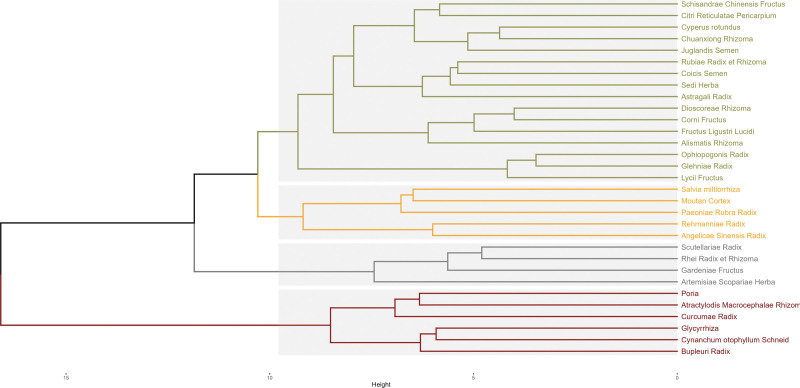
Cluster analysis of high-frequency TCMs (frequency ≥ 15 times). The dendrogram illustrates the results of a cluster analysis performed on 31 high-frequency TCMs used in the treatment of AIH, each with a frequency greater than or equal to 15. The Euclidean distance was employed as the measurement metric and Ward D method was used for clustering. The 31 TCMs can be categorized into 4 distinct clusters. AIH = autoimmune hepatitis, TCMs = traditional Chinese medicines.

A systematic clustering analysis on 31 high-frequency Chinese herbals (frequency ≥ 15) was performed using R language software, with the Euclidean distance as the measurement interval and Ward.D method. As shown in Figure 4, a total of 4 multi-drug combinations were obtained. Group C1 included *Corni Fructus, Dioscoreae Rhizoma, Fructus Ligustri Lucidi, Alismatis Rhizoma, Moutan Cortex, Angelicae Sinensis Radix, Cynanchum otophyllum Schneid*. Group C2 comprised *Citri Reticulatae Pericarpium, Schisandrae Chinensis Fructus, Cyperus rotundus, Chuanxiong Rhizoma, Juglandis Semen, Rubiae Radix et Rhizoma, Coicis Semen, Sedi Herba, Astragali Radix, Glehniae Radix, Ophiopogonis Radix, Lycii Fructus, Salvia miltiorrhiza, Paeoniae Rubra Radix*. Group C3 contained *Rhei Radix et Rhizoma, Scutellariae Radix, Artemisiae Scopariae Herba, Gardeniae Fructus.* Group C4 consisted of *Atractylodis Macrocephalae Rhizoma, Poria, Curcumae Radix, Glycyrrhiza, Cynanchum otophyllum Schneid, Bupleuri Radix*.

### 3.7. Network pharmacology analysis

#### 3.7.1. Screening of core drug, active ingredient and AIH-related target:

**Figure 5.**
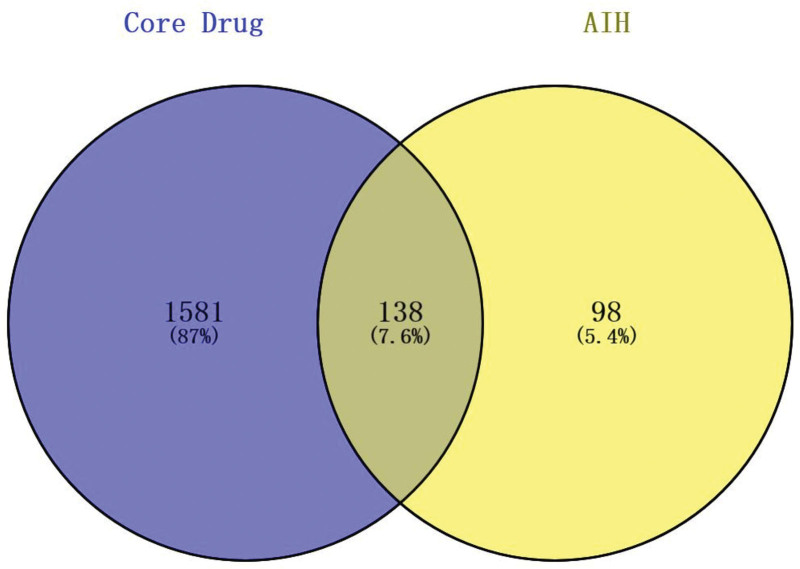
Prediction of potential targets for core drug therapy for AIH. The blue circles represent the core drug targets, the yellow circles represent the AIH targets, and there were 138 intersection targets, accounting for 7.6%. AIH = autoimmune hepatitis.

**Figure 6.**
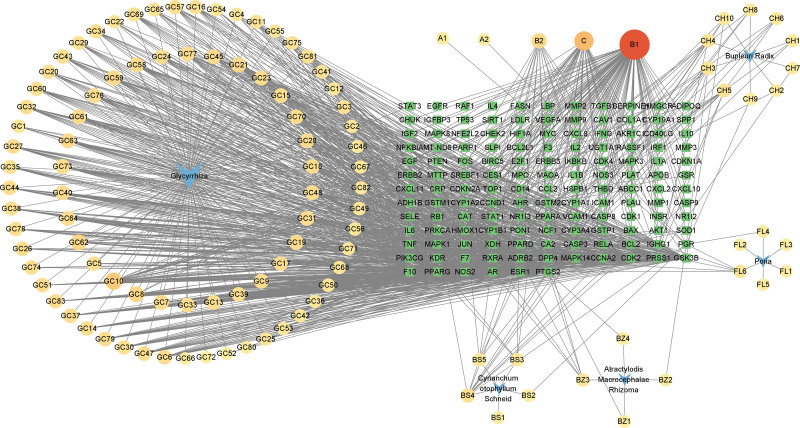
Drug-component-target network diagram. Blue arrows represent the core herbs, circular nodes represent the ingredients of the core herbs, and green square nodes represent the intersection targets of the herbs and disease (AIH) targets. Edges represent interactions between ingredients and targets. Among these, the orange-red node B (quercetin) connected with the most intersecting targets. AIH = autoimmune hepatitis.

According to the results of integrating frequency, association rule analysis, and clustering results, the “*Atractylodis Macrocephalae Rhizoma*-*Glycyrrhiza*-*Cynanchum otophyllum Schneid*-*Bupleuri Radix-Poria*” was determined to be the core drug combination. One hundred and thirty eight categories of active ingredients in the core drugs were obtained with the criteria of OB ≥ 30% and DL ≥ 0.18. After eliminating ingredients that do not have a target to point to, 113 ingredients were retained. The active ingredients in *Atractylodis Macrocephalae Rhizoma*, *Glycyrrhiza*, *Cynanchum otophyllum Schneid*, *Bupleuri Radix*, and *Poria* were 4, 88, 8, 13, and 6, respectively (listed in Table 11). Based on the result of target prediction, there were 23 targets for *Atractylodes macrocephala*, 1754 targets for *Glycyrrhiza*, 123 targets for *Paeonia lactiflora*, 348 targets for *Bupleurum*, and 22 targets for *Poria*, respectively. By searching GeneCards, OMIM, Disgenet, and Drugbank databases, 1719 AIH-related targets were obtained after merging and deleting duplicate targets. To determine the potential targets of AIH treated by core herbals, the targets associated with the selected active ingredients were intersected with AIH-related targets, and 138 common targets were obtained (Fig. 5). Then, the “core herbals-active ingredients-disease-related targets” network was constructed with Cytoscape 3.9.1 software (Fig. 6). There were 257 nodes included in this network. The circular nodes represent the active ingredient of core herbals, and the darker colors indicate higher degree values. The green rectangular nodes signify the docked targets, and the blue triangular nodes indicate the core herbals. There were 1153 edges in this network containing 1045 ingredient-target pair information. The top 5 compounds with the highest degree were quercetin, kaempferol, naringenin, licochalcone A, and formononetin, which may be the key active ingredients in treating AIH by the core herbals.

**Table 11:** Main active ingredients of core drug obtained from TCMSP database.

Core drug	Serial number	Ingredients	OB (%)	DL
*Cynanchum o tophyllum Schneid*	BS1	Paeoniflorgenone	87.59	0.37
	BS2	(3S,5R,8R,9R,10S,14S)-3,17-dihydroxy-4,4,8,10,14-pentamethyl-2,3,5,6,7,9-hexahydro-1H-cyclopenta[a]phenanthrene-15,16-dione	43.56	0.53
	BS3	Paeoniflorin	53.87	0.79
	BS4	Beta-sitosterol	36.91	0.75
	BS5	(+)-Catechin	54.83	0.24
*Glycyrrhiza*	GC1	Inermine	75.18	0.54
	GC2	DFV	32.76	0.18
	GC3	Glycyrol	90.78	0.67
	GC4	Jaranol	50.83	0.29
	GC5	Medicarpin	49.22	0.34
	GC6	Lupiwighteone	51.64	0.37
	GC7	7-Methoxy-2-methyl isoflavone	42.56	0.2
	GC8	Formononetin	69.67	0.21
	GC9	Calycosin	47.75	0.24
	GC10	Naringenin	59.29	0.21
	GC11	(2S)-2-[4-hydroxy-3-(3-methylbut-2-enyl)phenyl]-8,8-dimethyl-2,3-dihydropyrano [2,3-f] chromen-4-one	31.79	0.72
	GC12	Euchrenone	30.29	0.57
	GC13	Glyasperin B	65.22	0.44
	GC14	Glyasperin F	75.84	0.54
	GC15	Glyasperin C	45.56	0.4
	GC16	Isotrifoliol	31.94	0.42
	GC17	-1-(2,4-dihydroxyphenyl)-3-(2,2-dimethylchromen-6-yl)prop-2-en-1-one	39.62	0.35
	GC18	Kanzonols W	50.48	0.52
	GC19	(2S)-6-(2,4-dihydroxyphenyl)-2-(2-hydroxypropan-2-yl)-4-methoxy-2,3-dihydrofuro [3,2-g] chromen-7-one	60.25	0.63
	GC20	Semilicoisoflavone B	48.78	0.55
	GC21	Glepidotin A	44.72	0.35
	GC22	Glepidotin B	64.46	0.34
	GC23	Phaseolinisoflavan	32.01	0.45
	GC24	Glypallichalcone	61.6	0.19
	GC25	(6-hydroxy-2-benzofuranyl)-2,2-dimethyl-5-chromenol	58.44	0.38
	GC26	Licochalcone B	76.76	0.19
	GC27	licochalcone G	49.25	0.32
Continuation sheet				
Core drug	Serial number	Ingredients	OB (%)	DL
	GC29	Licoricone	63.58	0.47
	GC30	Gancaonin A	51.08	0.4
	GC31	Gancaonin B	48.79	0.45
	GC32	(3,4-dihydroxyphenyl)-5,7-dihydroxy-8-(3-methylbut-2-enyl)chromone	66.37	0.41
	GC33	5,7-dihydroxy-3-(4-methoxyphenyl)-8-(3-methylbut-2-enyl)chromone	30.49	0.41
	GC34	(3,4-dihydroxyphenyl)-5,7-dihydroxy-6-(3-methylbut-2-enyl)chromone	44.15	0.41
	GC35	Glycyrin	52.61	0.47
	GC36	Licocoumarone	33.21	0.36
	GC37	Licoisoflavone	41.61	0.42
	GC38	Licoisoflavone B	38.93	0.55
	GC39	licoisoflavanone	52.47	0.54
	GC40	shinpterocarpin	80.3	0.73
	GC41	(E)-3-[3,4-dihydroxy-5-(3-methylbut-2-enyl)phenyl]-1-(2,4-dihydroxyphenyl)prop-2-en-1-one	46.27	0.31
	GC42	Liquiritin	65.69	0.74
	GC43	Licopyranocoumarin	80.36	0.65
	GC44	Glyzaglabrin	61.07	0.35
	GC45	Glabridin	53.25	0.47
	GC46	Glabranin	52.9	0.31
	GC47	Glabrene	46.27	0.44
	GC48	Glabrone	52.51	0.5
	GC49	1,3-dihydroxy-9-methoxy-6-benzofurano[3,2-c]chromenone	48.14	0.43
	GC50	1,3-dihydroxy-8,9-dimethoxy-6-benzofurano [3,2-c]chromenone	62.9	0.53
	GC51	Eurycarpin A	43.28	0.37
	GC52	(−)-Medicocarpin	40.99	0.95
	GC53	Sigmoidin-B	34.88	0.41
	GC54	(2R)-7-hydroxy-2-(4-hydroxyphenyl)chroman-4-one	71.12	0.18
	GC55	(2S)-7-hydroxy-2-(4-hydroxyphenyl)-8-(3-methylbut-2-enyl)chroman-4-one	36.57	0.32
	GC56	Isoglycyrol	44.7	0.84
	GC57	Isolicoflavonol	45.17	0.42
	GC58	HMO	38.37	0.21
	GC59	1-Methoxyphaseollidin	69.98	0.64
	GC60	Quercetin der.	46.45	0.33
	GC61	3’-Hydroxy-4’-O-Methylglabridin	43.71	0.57
Continuation sheet				
Core drug	Serial number	Ingredients	OB (%)	DL
	GC62	Licochalcone a	40.79	0.29
	GC63	3’-Methoxyglabridin	46.16	0.57
	GC64	2-[(3R)-8,8-dimethyl-3,4-dihydro-2H-pyrano[6,5-f]chromen-3-yl]-5-methoxyphenol	36.21	0.52
	GC65	Inflacoumarin A	39.71	0.33
	GC66	Icos-5-enoic acid	30.7	0.2
	GC67	Kanzonol F	32.47	0.89
	GC68	6-Prenylated eriodictyol	39.22	0.41
	GC69	7,2’,4’-trihydroxy-5-methoxy-3-Arylcoumarin	83.71	0.27
	GC70	7-Acetoxy-2-methylisoflavone	38.92	0.26
	GC71	8-Prenylated eriodictyol	53.79	0.4
	GC72	Gadelaidic acid	30.7	0.2
	GC73	Vestitol	74.66	0.21
	GC74	Gancaonin G	60.44	0.39
	GC75	Gancaonin H	50.1	0.78
	GC76	Licoagrocarpin	58.81	0.58
	GC77	Glyasperins M	72.67	0.59
	GC78	Glycyrrhiza flavonol A	41.28	0.6
	GC79	Licoagroisoflavone	57.28	0.49
	GC80	Odoratin	49.95	0.3
	GC81	Phaseol	78.77	0.58
	GC82	Xambioona	54.85	0.87
	GC83	Dehydroglyasperins C	53.82	0.37
*Atractylodis Macrocephalae Rhizoma*	BZ1	14-Acetyl-12-senecioyl-2E,8Z,10E-atractylentriol	63.37	0.3
	BZ2	(3S,8S,9S,10R,13R,14S,17R)-10,13-dimethyl-17-[(2R,5S)-5-propan-2-yloctan-2-yl]-2,3,4,7,8,9,11,12,14,15,16,17-dodecahydro-1H-cyclopenta[a]phenanthren-3-ol	36.23	0.78
	BZ3	3β-Acetoxyatractylone	54.07	0.22
	BZ4	8β-Ethoxy atractylenolide Ⅲ	35.95	0.21
*Bupleuri Radix*	CH1	Linoleyl acetate	42.1	0.2
	CH2	Baicalin	40.12	0.75
	CH3	Stigmasterol	43.83	0.76
	CH4	3,5,6,7-tetramethoxy-2-(3,4,5-trimethoxyphenyl)chromone	31.97	0.59
	CH5	Areapillin	48.96	0.41
	CH6	Cubebin	57.13	0.64
	CH7	Longikaurin A	47.72	0.53
	CH8	(+)-Anomalin	46.06	0.66
	CH9	α-spinasterol	42.98	0.76
	CH10	Petunidin	30.05	0.31
*Poria*	FL1	Trametenolic acid	38.71	0.8
Continuation Sheet				
Core drug	Serial Number	Ingredients	OB (%)	DL
*Poria*	FL2	(2R)-2-[(3S,5R,10S,13R,14R,16R,17R)-3,16-dihydroxy-4,4,10,13,14- pentamethyl-2,3,5,6,12,15,16,17-octahydro-1H-cyclopenta[a]phenanthren-17-yl]-6-methylhept-5-enoic acid	30.93	0.81
	FL3	Cerevisterol	37.96	0.77
	FL4	Ergosta-7,22E-dien-3beta-ol	43.51	0.72
	FL5	Ergosterol peroxide	40.36	0.81
	FL6	Hederagenin	36.91	0.75
*Cynanchum otophyllum Schneid-Glycyrrhiza*	A1	Mairin	55.38	0.78
	A2	Sitosterol	36.91	0.75
*Glycyrrhiza-Bupleuri Radix*	B1	Quercetin	46.43	0.28
	B2	Isorhamnetin	49.6	0.31
*Cynanchum otophyllum Schneid-Glycyrrhiza-Bupleuri Radix*	C	Kaempferol	41.88	0.24

#### 3.7.2. Construction of PPI network:

**Figure 7.**
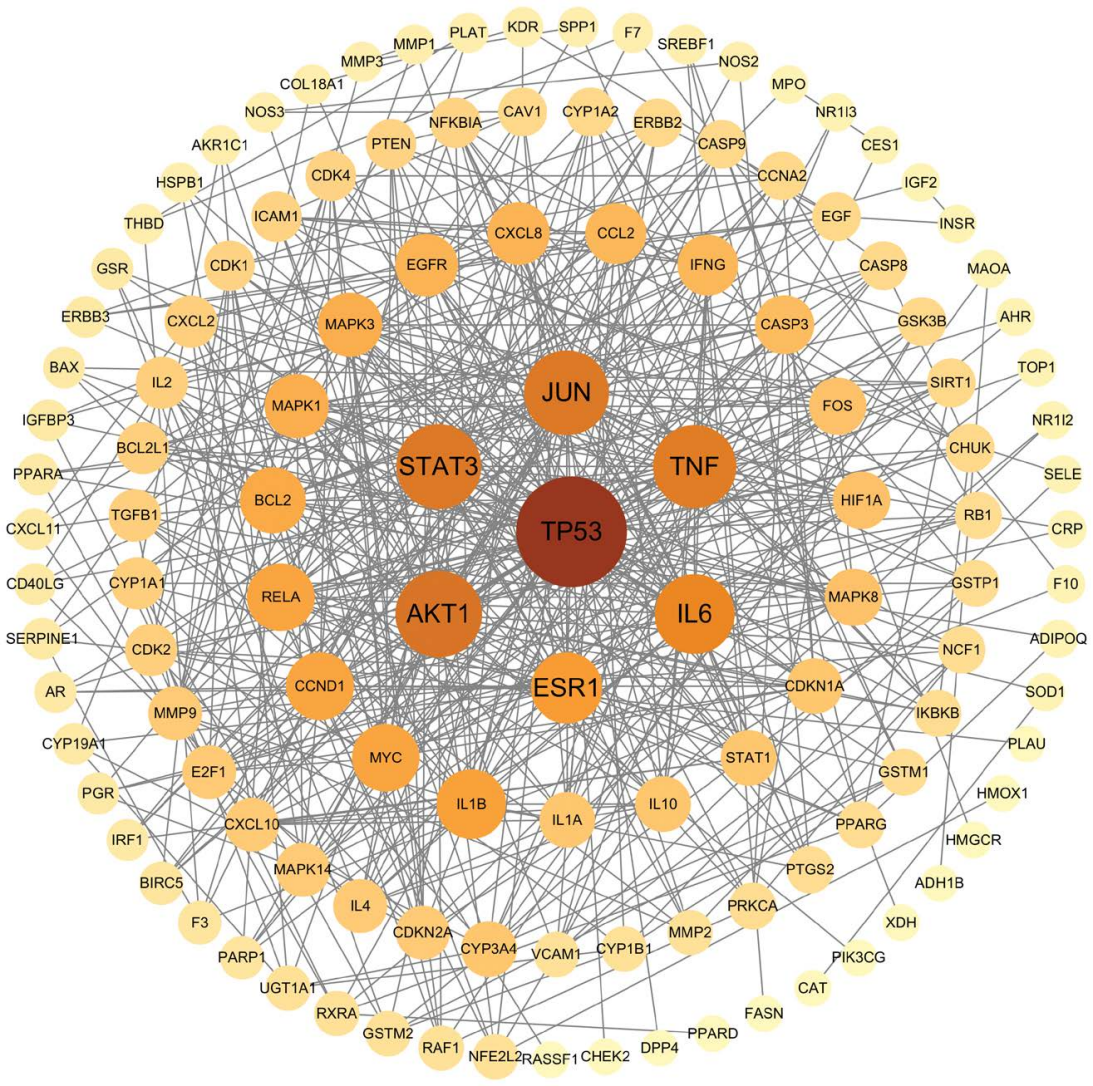
Protein–protein interaction network for core drug combinations to treat AIH. Circular nodes represent core drugs and core targets of AIH. The size and color depth of the nodes are proportional to the degree value of the targets. The larger and deeper the node, the higher the degree value. AIH = autoimmune hepatitis.

The interaction information between intersecting targets from the STRING database was imported into CytoScape 3.9.1 software for PPI network construction. As displayed in Figure 7, there were 124 nodes and 504 edges in this network, and the larger and darker nodes indicate closer interactions with other targets. With a degree value ≥ 25 as the screening criterion, 6 key targets, including TP53, AKT1, JUN, STAT3, TNF, and IL-6, were filtered out.

#### 3.7.3. GO and KEGG analysis:

**Figure 8.**
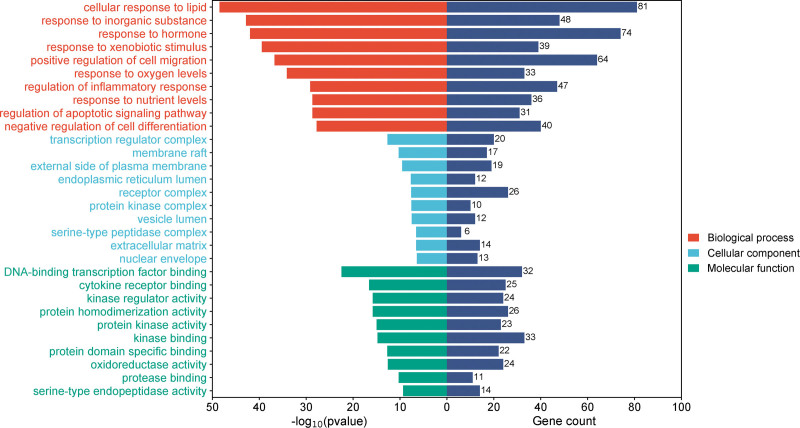
GO enrichment analysis. The bars on the left represent the top 10 GO terms for BP in green, CC in orange, and MF in light blue. The bars on the right indicate the number of genes associated with each GO term. BP = biological process, CC = cellular component, GO = gene ontology, MF = molecular function.

A total of 2024 items reflecting the biological functions of key targets of the core herbals were obtained by GO functional enrichment analysis, including 1790 items in the biological processes (BPs) category, 79 items in the cellular components category (CC), and 155 items in the molecular functions (MFs) items. The top 10 items with the *P*-value in ascending order involved in BP, CC, and MF categories were visualized in Figure 8. The items with the most significant statistical differences in BP, CC, and MF categories are cellular responses to lipids, transcription regulatory complexes, and DNA-binding transcription factor binding, respectively. The gene count in each item is also displayed in Figure 8.

**Figure 9.**
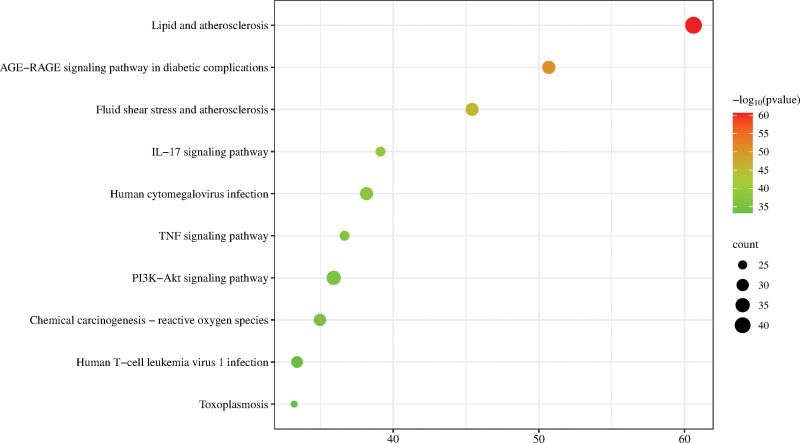
KEGG pathway enrichment analysis. The X-axis and Y-axis represent the ratio of genes involved in each process and the full names. The color and size of each bubble correspond to the *P*-value and gene count. KEGG = Kyoto encyclopedia of genes and genomes.

About 189 signaling pathways were screened out by KEGG enrichment analysis and the top 10 signaling pathways with the *P*-value in ascending order, except for the signaling pathways involved in all kinds of cancer, were selected for visualization in Figure 9, mainly including the IL-17 signaling pathway, TNF signaling pathway, and PI3K-Akt signaling pathway, etc.

## 4. Discussion

Because the pathogenesis of autoimmune hepatitis is not fully understood, corticosteroids and azathioprine are still the recommended first-line treatments for AIH.^[3]^ However, long-term therapy with these drugs may lead to serious complications including steroid-induced diabetes, osteoporosis, avascular necrosis, psychiatric symptoms, hypertension, and cataracts.^[17]^ Under the guidance of dynamic and holistic concepts in TCM, various academic genres have accumulated extensive treatment experiences for treating AIH by adopting different therapeutic strategies and Chinese medicinal compositions. Nevertheless, due to the lack of detailed descriptions of the relationship between treatment outcomes, methodologies, and Chinese herbs, the translation of experiential knowledge into an effective diagnostic and treatment system for AIH with TCM has not been realized. With the development of modern bioinformatics technologies, data mining, and network pharmacology assay provide powerful tools for effectively extracting and processing information for TCM applied to treat AIH.

TCM does not have a precise definition for AIH but has documented its clinical manifestations, such as jaundice, hypochondriac pain, and distention and fullness. Nowadays, scholars of TCM classify the etiology and pathogenesis of AIH as the deficiency in origin and excess in superficial symptoms. The deficiency is mainly Qi deficiency and Yin deficiency of the liver and kidney, while the excess is mostly shown as qi stagnation, dampness-heat, dampness-turbid, and blood stasis.^[2]^ According to the analysis in this article, it was found that most of the commonly used herbs in TCM compound prescriptions for treating AIH are cool in property, sweet and bitter in flavor, and attribution to the liver meridian. The P Pearson correlation analysis was used to evaluate the association between the Chinese herbals. The higher the correlation coefficient, the stronger the association and the higher the potential of the combined application. However, because the correlation analysis did not consider the frequency of use of the single Chinese herbal, the correlation analysis could not fully reflect the combination mode of Chinese medicine in the treatment of AIH, and the correlation analysis conclusions were for reference only. For example, *Saposhnikoviae Radix* and *Schisandrae Chinensis Fructus* have the highest correlation (correlation coefficient is 0.65). However, its application times were only 12 and 25, so it is not used as the preferred herb pair in AIH treatment practice. Association rule analysis reveals that {*Atractylodis Macrocephalae Rhizoma*} => {*Bupleuri Radix*}, {*Cynanchum otophyllum Schneid*, *Atractylodis Macrocephalae Rhizoma*}=>{*Bupleuri Radix*}, and {*Cynanchum otophyllum Schneid*, *Glycyrrhiza*, *Poria*, *Atractylodis Macrocephalae Rhizoma*}=>{*Bupleuri Radix*} are the combinations with the highest support in the 2, 3, 4, and 5-herb association rules, respectively. Moreover, *Bupleuri Radix*, *Atractylodes macrocephala* Koidz, and *Cynanchum otophyllum Schneid* are the most frequently associated herbs indicated by the bubble charts. Four drug combinations were obtained by cluster analysis, including Liuwei Dihuang Pill plus or minus, nourishing Yin and Qi and activating blood combination, Yinchenhao Decoction plus flavor, and Xiaoyao San minus flavor. Based on the results of frequency of use, association rule analysis, and cluster analysis, the “*Atractylodes macrocephala Koidz.*- *Glycyrrhizae Radix et Rhizoma* - *Cynanchum otophyllum Schneid* - *Bupleuri Radix* - *Poria cocos (Schw.) Wolf.*” combination was chosen as the core formula, which essentially is a Xiaoyao San minus flavor. According to the basic Chinese medicine theory, *Bupleuri Radix* relieves liver qi stagnation, *Cynanchum otophyllum Schneid* with its sour and slightly cold properties nourishes the blood and yin, and soothes the liver. *Cynanchum otophyllum Schneid* combined with *Bupleuri Radix* can invigorate the liver function by tonifying the substance and making the liver smooth and steady by filling and harmonizing the blood. *Atractylodes macrocephala Koidz*, *Poria cocos (Schw.) Wolf.*, and *Glycyrrhizae Radix et Rhizoma* strengthen the spleen and augment qi to maintain the stability of the Earth element by governing the Wood element so that to ensure the generation of nutrient-blood.

It has been reported that Xiaoyao San has demonstrated efficacy in clinical use for AIH.^[18,19]^ *Bupleurum*, with around 74 compounds including essential oils, triterpenoid saponins, and polyacetylenes,^[20]^ has its triterpenoid saponins identified as the main bioactive components responsible for its primary pharmacological effects.^[21,22]^ These saponins have been extensively studied for their analgesic, immunomodulatory, hepatoprotective, and anti-inflammatory properties. Notably, research by Chun et al underscores the anti-inflammatory efficacy of *Bupleurum* saponins through mechanisms such as inhibition of exudation, capillary permeability, mediator release, leukocyte migration, and connective tissue proliferation, alongside mitigating various allergic inflammations.^[23]^ Additionally, these saponins have demonstrated hepatoprotective capabilities by modulating intracellular calcium levels and significantly diminishing levels of liver enzymes and lipid peroxidation in rat models of acute liver damage induced by carbon tetrachloride (CCl4).^[24,25]^ *Atractylodis Macrocephalae Rhizoma*, with approximately 170 isolated chemical components spanning volatile oils, polysaccharides, lactones, vitamins, amino acids, and resins, places lactones and polysaccharides at the forefront of its bioactive constituents. Atractylodis Lactone I and III, particularly, are prominent for their anti-inflammatory action through the marked inhibition of tumor necrosis factor-alpha and nitric oxide production. Moreover, Atractylodis polysaccharides have showcased a broad spectrum of biological activities, including enhancing immune function and alleviating viral liver damage, highlighting their vital role in the medicinal utility of *Atractylodis Macrocephalae Rhizoma*.^[26]^ *Glycyrrhiza* harbors an array of compounds such as triterpenoid saponins, flavonoids, coumarins, and alkaloids,^[27]^ with glycyrrhizic acid particularly noteworthy for its role in regulating the expression of cytochrome P450 enzymes CYP3A and CYP7A. This regulation is pivotal in reducing levels of liver enzymes in rats, and enhancing antioxidant levels to protect mouse livers from cholic acid-induced damage.^[28,29]^ Polysaccharides are the most abundant component of *Poria*. It exhibits a wide range of biological activities including immunomodulation, anti-inflammatory, and anti-hepatitis effects, and has extensive therapeutic applications in TCM practices.^[30]^ Furthermore, the total glucosides of peony, identified as the primary pharmacologically active ingredient in *Cynanchum otophyllum Schneid*, are celebrated for their significant hepatoprotective properties. These glucosides are effective against a variety of liver conditions, including acute liver injury, nonalcoholic fatty liver disease, chronic liver fibrosis, and liver cancer, thereby reinforcing the medicinal value of these compounds in TCM.^[31]^

Further network pharmacology studies have revealed the active ingredients of the core TCM formula “ Atractylodes macrocephala Koidz.- Glycyrrhizae Radix et Rhizoma - Cynanchum otophyllum Schneid - Bupleuri Radix - Poria cocos (Schw.) Wolf.” for treating AIH, including quercetin, kaempferol, naringenin, licochalcone A, and formononetin, and TP53, AKT1, JUN, STAT3, TNF, and IL-6 may be the key targets for these medicinal compositions. Quercetin, with the highest degree value, is a flavonoid abundant in vegetables and fruits. It has anti-inflammatory, antimicrobial, antioxidant, and analgesic properties, and it has been proven quercetin can protect mice from Concanavalin (Con) A-induced liver injury by down-regulating the HMGB1-TLR and the NF-κB signaling pathway. We also have proved that quercetin can protect liver cells from apoptosis and ferroptosis induced by macrophage in vitro (presented in another paper).

Quercetin exhibits antioxidant, anti-inflammatory, and immunomodulatory effects.^[32]^ Quercetin, a major bioactive component of Danggui Buxue Decoction, has been demonstrated to interact with critical targets like p53, IL-6, and AKT1, consequently ameliorating premature ovarian failure by regulating the estrogen receptor/androgen receptor balance in the TP53-AKT signaling cascade.^[33]^ Additionally, quercetin, a key active component of Yinchenhao Decoction, exhibits novel anti-inflammatory properties. Studies have shown that quercetin mitigates macrophage-induced hepatocyte injury by reducing inflammatory responses, apoptosis, and ferroptosis, highlighting its potential therapeutic role in AIH-related liver damage.^[34]^ Kaempferol, a flavonoid initially identified in Camellia sinensis^[35]^ and found in diverse plant species,^[36]^ displays anti-inflammatory,^[37]^ hepatoprotective,^[38,39]^ and antioxidant properties.^[40]^ Ling et al demonstrated kaempferol can modulate the expression of JUN and AKT1, and decrease circulating levels of IL-6 and TNF-α, thereby exerting anti-inflammatory effects.^[41]^ Naringenin, a flavonoid abundant in grapefruits and other citrus fruits, possesses anti-inflammatory, antioxidant, and hepatoprotective properties.^[42]^ In a diethylnitrosamine (DEN) and 2-acetylaminofluorene (2AAF)-induced mouse model of hepatocarcinogenesis, supplementation with quercetin and naringenin markedly attenuated the inflammatory cell infiltration in liver and neoplastic lesions, potentially by regulating p53 expression, thereby eliciting anti-inflammatory and pro-apoptotic responses.^[43]^ Formononetin, a phytoestrogen belonging to the flavonoid class, displays antioxidant, antihypertensive, antineoplastic, and antimicrobial properties.^[44]^ In a rat model of cerebral ischemia-reperfusion injury, formononetin was found to markedly ameliorate neurological impairments and brain tissue damage, possibly through suppressing the expression of JAK2/STAT3 pathway, and consequently decreasing levels of inflammatory mediators such as TNF-α and IL-6, thereby conferring anti-inflammatory effects and neuroprotection.^[45]^

GO enrichment analysis has shown that mechanism of the core TCM formula “Atractylodes macrocephala Koidz.-Glycyrrhizae Radix et Rhizoma-Cynanchum otophyllum Schneid-Bupleuri Radix-Poria cocos (Schw.) Wolf.” in treating AIH mainly involved in the BPs such as cellular response to lipids, response to inorganic substances, response to hormones, response to xenobiotic stimuli, and positive regulation of cell migration. In terms of CCs, this combination is primarily localized to transcription regulatory complexes, membrane rafts, the outer side of the plasma membrane, the endoplasmic reticulum lumen, and receptor complexes. Furthermore, the MF related to this core TCM formula involved in DNA-binding transcription factor binding, cytokine receptor binding, kinase regulator activity, homotypic dimer activity, and protein kinase activity. KEGG enrichment analysis indicates that the targets regulated by the core TCM formula “ Atractylodes macrocephala Koidz.-Glycyrrhizae Radix et Rhizoma-Cynanchum otophyllum Schneid-Bupleuri Radix-Poria cocos (Schw.) Wolf.” are mainly enriched in the IL-17 signaling pathway, TNF signaling pathway, and PI3K-Akt signaling pathway. It was indicated that it is possible to discover new specific drugs for treating AIH by further exploring the intrinsic relationship between these pharmacodynamic components and these signaling pathways or BPs.

## 5. Conclusion

Based on our research with in-depth data mining and network pharmacology analysis, the core TCM formula “Atractylodes macrocephala Koidz.-Glycyrrhizae Radix et Rhizoma-Cynanchum otophyllum Schneid-Bupleuri Radix-Poria cocos (Schw.) Wolf.” may have a good potential and unique advantages in the treatment of AIH. The related medicinal compositions, targets, signaling pathways, and BPs of this core TCM formula can provide valuable data support for future clinical and basic research. This discovery not only offers a new idea for the treatment of AIH by TCM but also brings more accurate and efficient treatment options for patients. It is hoped that through further research, a systematic treatment system for TCM on AIH can be formed, and more comprehensive and in-depth treatment programs can be supplied for patients with AIH or susceptible populations.

## 6. Formatting of funding sources

This study was funded by the construction project of high-level TCM key discipline of National Administration of TCM (2024); Key team of scientific and technological innovation talents of Shanxi Province with integrated traditional Chinese and Western medicine for preventing and treating rheumatological diseases (Grant Number: 202204051002033); Special project with important nations of scientific and technological cooperation and exchange of Shanxi Province (Grant Number: 202104041101013); Key laboratory of rheumatological and immunological diseases treated by integrated Chinese and Western medicine (zyyyjs2024021); Shanxi Province science foundation of free exploration category (202203021222272); Scientific research project of Administration of TCM in Shanxi Province (2024ZYYAD008); Science and technology innovation project of Shanxi University of Chinese Medicine (Grant Number: 2022TD2003).

## Author contributions

**Investigation:** Di Guo, Xin Li, Shiya Wei, Fenqing Cai.

**Supervision:** Yang Liu.

**Writing – original draft:** Di Guo.

**Writing – review & editing:** Yang Liu.
